# The association between visceral fat obesity and prefrailty in Chinese older adults: a cross-sectional study

**DOI:** 10.1186/s12902-024-01625-1

**Published:** 2024-08-02

**Authors:** Yue Wang, Suxing Shen, Peipei Han, Kai Zheng, Cheng Chen, Yahui Wu, Chuanjun Huang, Jiangling Guo, Yiqiong Qi, Xiaoyu Chen, Yuxuan Zheng, Xinwei Xia, Siyan Peng, Qi Guo

**Affiliations:** 1 School of Health Science and Engineering, University of Shanghai for Science and Technology, Shanghai, 200135 China; 2 Rehabilitation Medicine Department, Tianjin Fifth Central Hospital, Tianjin, 300457 China; 3 Shanghai University of Medicine and Health Sciences, Shanghai, 201318 China; 4 School of Health, Fujian Medical University, Fuzhou, 350122 Fujian China; 5 Graduate School of Shanghai University of Traditional Chinese Medicine, Shanghai University of Traditional Chinese Medicine, Shanghai, 201203 China; 6 Department of Sport Rehabilitation, Shanghai University of Sport, Shanghai, 200438 China; 7 Department of Rehabilitation Medicine, Shanghai University of Medicine and Health Sciences affiliated Zhoupu Hospital, 279 Zhouzhu Highway, Pudong New Area, Shanghai, 201318 China

**Keywords:** Prefrailty, Visceral fat area, Obesity, Older adults

## Abstract

**Background:**

The prevalence of obesity is escalating. Previous research has concentrated on the link between frailty and obesity; however, the association between prefrailty and obesity has been less studied. Prefrailty screening and intervention may prevent or postpone frailty in older persons.

**Objective:**

The study was to investigate into the relationship between prefrailty and several obesity indicators in Chinese community-dwelling older individuals.

**Methods:**

This research employed the Frailty Screening Index to investigate the frailty phenotype of people living in Shanghai. Bioelectrical impedance analysis was used for evaluating body composition.

**Results:**

There were 510 participants (39.0%) with high visceral adipose areas. Participants with a high visceral adipose area showed a higher risk of prefrailty (adjusted OR, 1.53; 95% CI, 1.19–1.96), according to multivariate models. When body mass index (BMI) and visceral fat area (VFA) were combined, it was discovered that having an overweight BMI with normal VFA was a protective factor for prefrailty (corrected OR, 0.62; 95% CI, 0.43–0.90), but having a normal weight but excess VFA increased the risk of prefrailty (corrected OR, 1.87; 95% CI, 1.15–3.03).

**Conclusion:**

Visceral fat obesity is an independent risk factor for prefrailty in Chinese older adults. Implementing targeted interventions, such as dietary modifications, increased physical activity, and other lifestyle changes, could play a crucial role in reducing the risk of prefrailty and improving overall health outcomes in this population.

## Introduction

Frailty is a multisystem medical syndrome of decreased function characterized by increased susceptibility to stressful conditions and an elevated risk of impairment, falls, fractures, hospitalization, and death [1]. Prefrailty, a condition that may be curable before the advent of established frailty, is a prodromal stage that is connected to frailty [2]. In China, Fan et al. found a 40% prevalence of prefrailty among Chinese adults aged 30–79, with higher rates in older individuals (aged 65 and above) and those with unhealthy lifestyles [3]. It is worth noting that the risk of death among those with prefrailty is 50% higher than that of non-frail older adults [4, 5], yet little is known about prefrailty. The advent of coronavirus disease 2019 (COVID-19) over the previous two years has had a significant impact on the general public’s daily life [6].This has resulted in a decrease in physical activity, muscle mass, and increased body fat [7, 8], and possibly accelerated frailty. Therefore, improved understanding of prefrailty is important for the prevention and management of frailty.

Obesity and overweight are defined as an excessive accumulation of fat, which increases the risk of adverse health outcomes. During the COVID-19 pandemic, obesity indicators such as BMI, waist circumference and WHR increased significantly among middle-aged and older Chinese individuals [9]. Therefore, obesity deserves more attention. Obese older adults have been reported to have a higher risk of frailty [10]. It is generally believed that obesity can contribute to insulin resistance, oxidative stress, and inflammation, which all lead to frailty [11, 12]. The precise connection between obesity and frailty has been debated in other studies. According to some research, frailty is much more common among obese older persons [13, 14]. However, it has also been demonstrated that frail women who are overweight or obese have a reduced risk of clinical adverse events [15]. The obesity paradox, a notable “obesity paradox” has emerged, indicating that overweight and obese individuals with cardiovascular conditions exhibit prolonged survival times relative to their counterparts with a normal BMI in recent times [16], also applies to individuals with frailty [17, 18].

Frailty is associated with the type of fat distribution. Some studies have also shown that there is a benign metabolic phenotype of fat and that obesity is not necessarily unhealthy. This may explain the paradox that some obese people have a low risk of disease [19]. The health implications are influenced by various factors including the distribution of fat in the body and the body’s ability to adapt to excess caloric intake [20]. Generally, BMI is considered to represent generalized obesity, while abdominal obesity is associated with metabolic diseases. Widely accepted is that the obesity paradox is applicable to older individuals, as central adiposity is not taken into consideration due to the failure to account for central adiposity [21]. Although previous research indicated that BMI and central obesity indicators are linked to a higher risk of frailty [10], previous studies have given less attention to prefrailty, so it is unclear how these indicators are specifically associated with prefrailty. Understanding the relationship between different obesity types and pre-frailty could help determine which types of obesity are of greater concern and identify potential points of intervention to prevent prefrailty. Therefore, the purpose of this study was to investigate the relationship between body mass index (BMI), central obesity, and prefrailty in older adults in Shanghai.

## Methods

### Study design and population

This research was cross-sectional and descriptive. The survey population was recruited in 2021 among a total of 1405 older adults in the community in two regions, Chongming and Hongkou of Shanghai. All subjects were invited to participate in a free national medical screening program. Individuals were excluded based on the following criteria: (1) incapacity to complete body composition or physical fitness tests; (2) incapacity to converse with the research staff or give informed consent; and (3) severe cognitive impairment or mental illness. A standardized questionnaire was utilized by trained researchers to collect information for the study. The final study population consisted of 1368 individuals, all of whom had given their informed consent prior to taking part.

### Demographics and covariates

Interview questionnaires were administered by trained research staff and included sociodemographic information(gender, age, marital status, and level of education), behavioural traits (smoking and drinking behaviors), and medical condition information as previously mentioned [22, 23]. The IPAQ short form was employed to measure physical activity [24]. The 30-item Geriatric Depression Scale (GDS-30) was used to identify depression; a score of less than 11 was regarded as a diagnosis of depression [25]. Additionally, we also investigated whether participants had chronic health conditions, such as type-2 diabetes mellitus (T2DM), hypertension, hyperlipidaemia, cardiovascular disease (CVD) and stroke. Details of the survey methodology were described in our previous cross-sectional study [23].

### Frailty

The Frailty Screening Index, a modified screening tool based on the Cardiovascular Health Study criteria [26], was employed to assess frailty phenotypes. It is noteworthy that, to make up for the diminished body size of an East Asian population, a weight loss of 3 kg was implemented instead of 5 kg. The frailty screening index consists of the following five items: (1) Unintentional weight loss of 3.0 kg or 5% of body weight in the previous year was regarded as shrinking; (2) Weakness was assessed with the handgrip strength threshold established for Chinese older individuals, adjusted for sex and BMI; (3) Fatigue was determined by two questions from the Center for Epidemiological Studies Depression Scale; (4) Slowness used the cut-off value of walking speed, adjusted for sex and height; and (5) Inactivity was defined as Based on the Taiwan International Physical Activity Questionnaire Short Form (IPAQ-SF). The weekly energy expenditure for activities with a metabolic equivalent (MET) of 2 or lower was found to be 383 kcal for men and 270 kcal for women. Frailty was defined as meeting three or more of these criteria, while prefrailty was identified as meeting one or two of these criteria.

### Anthropometric anthropometry

BIA is now a straightforward and practical diagnostic method for determining body composition. We assessed body composition using bioelectrical impedance analysis (BIA) (Inbody770, Biospace, Korea) after measuring height and weight. During the measurement, participants without pacemakers were in a standing position, with arms naturally hanging down, thighs not touching each other but extended to shoulder width. Participants’ height and weight were measured at inclusion. Visceral fat obesity was defined as VFA ≥ 100 [27].

By dividing weight (kg) by the square of height (m2), the BMI was calculated. Individuals were categorized as underweight, normal weight, overweight, and obese (18.5, 18.5–24.0, 24.0-27.9, 28.0 kg/m2) based on The Cooperative Meta-Analysis Group of China Obesity Task Force’s criteria [28]. Abdominal obesity was defined as WC > 85 cm in men and > 80 cm in women [29]. An inelastic measuring tape was used to measure the WC at the midpoint of the iliac crests and rib margin in a horizontal plane. Additionally, well-trained investigators completed the entire measurement.

### Statistical analysis

For continuous variables, we used mean and median values and standard deviations, and proportions were used for categorical variables. With the Bonferroni correction, the independent samples t-test was used to examine differences between continuous variables. Categorical variables were subjected to the chi-square test. The relationship between body mass index and other signs of central obesity and frailty was evaluated using logistic regression models with asymptotic levels of adjustment. Age and sex were the factors taken into account for adjustment (Model 1). Model 2 was modified to account for factors such as gender, age, alcohol and tobacco use, education, depression, widowhood, living alone, and chronic illnesses (diabetes, heart disease, and stroke). All statistical analyses were performed using IBM SPSS v25.0 software (SPSS Inc., Chicago, IL, USA). The significance level in the current research was set as α = 0.05.

## Results

### Baseline study participant characteristics

From two centres in Shanghai, a total of 1,405 participants were recruited. The questionnaires and measurements were completed by 1368 older residents of the community. After excluding 60 frail participants, there were 651 (49.8%) prefrail persons as shown in Table 1. The participants’ average age was 72.3 years, and 56.7% (740) of them were female. Regarding the obesity indicators, the prefrail group had higher BMI, VFA and waist circumference than the robust group, but only VFA was statistically different between the two groups. More people in the prefrail group were widowed, lived alone, suffered from depression, diabetes, heart disease, and stroke and were less educated, compared to the robust group. No significant differences were found between the two groups in terms of alcohol consumption, tobacco use, hyperlipidaemia, and hypertension.

**Table 1:** The characteristics by pre-frailty and robust

Variables	Total (*N* = 1308)	Prefrail (*N* = 651)	Robust (*N* = 657)	*P* value
**Demographics**				
Age(year)	72.3 ± 5.7	73.7 ± 6.3	70.9 ± 4.5	< 0.001*
**Sex, n (%)**				0.099
Female	741(56.7)	354(54.4)	387(58.9)	
Male	567(43.3)	297(45.6%)	270(41.1)	
**Education (%)**				0.002*
Illiteracy	39(3.0)	30(4.6)	9(1.4)	
Primary school	245(18.7)	117(18.0)	128(19.5)	
Middle school and above	1024(78.3)	504(77.4)	520(79.1)	
Widowed, n (%)	184(14.1)	109(16.8)	75(11.4)	0.005*
Living alone, n (%)	165(12.6)	97(14.9)	68(10.4)	0.014*
Depression, n (%)	154(11.8)	25.36	23.14	0.000*
**Smoking (%)**				0.787
Never	981(75.2)	484(74.7)	497(75.8)	
Former	176(13.5)	87(13.4)	89(13.6)	
Current	147(11.3)	77(11.9)	70(10.7)	
**Drinking (%)**				0.906
Never	868(66.6)	436(67.4)	432(65.8)	
Former	118(9.0)	59(9.0)	59(9.1)	
Sometimes	226(17.3)	108(16.7)	118(18.0)	
Everyday	92(7.1)	48(7.3)	44(6.8)	
**Obesity indicators**				
BMI (kg/m^2^)	23.91 ± 3.28	23.93 ± 3.56	23.89 ± 2.97	0.842
VFA (cm^2^)	94.88 ± 34.49	97.61 ± 37.32	92.55 ± 32.07	0.008*
WC (cm)	84.38 ± 9.84	84.80 ± 10.12	83.96 ± 9.55	0.122
**Chronic conditions, n (%)**				
Diabetes	293(22.4)	179(27.5)	114(17.4)	< 0.001*
Hypertension	856(65.4)	438(67.3)	418(63.6)	0.164
Hyperlipidemia	625(47.8)	319(49.1)	306(46.6)	0.365
Stroke	209(16.0)	124(19.0)	85(13.0)	0.003*
Heart disease	239(18.3)	138(21.2)	101(15.4)	0.006*

*Abbreviations* BMI Body Mass Index, VFA Visceral Fat Area, WC Waist Circumstance

*P < 0.05

### The association between the risk of prefrailty and obesity indicators

The ratios of BMI, WC, and VFA to prefrailty risk, with their 95% confidence intervals, are presented in Table 2 with a significantly greater risk of prefrailty for those with visceral obesity than for leaner individuals, after age and sex were taken into account [OR (95% CI) of 1.47(1.15,1.86)]. Adjusting for drinking, smoking, education, depression, widowed status, living alone, and chronic diseases (diabetes, stroke, heart disease) (Model 2), a significantly higher risk for prefrailty was still associated with visceral obesity, with an OR (95% CI) of 1.53(1.19,1.96)]. We then further investigated the relationship between obesity, defined by other indicators, and the odds of prefrailty. Adjusting for Model 2, no longer was obesity, as defined by BMI [OR (95% CI) of 0.97(0.77,1.23)] and waist circumference [OR (95% CI) of 0.95(0.74,1.20)]], associated with an increased risk of prefrailty.

**Table 2:** Associations between the three definitions of obesity and odds of pre-frailty

Variables	*N* (%)	Crude OR (95%CI)	Model1 OR (95%CI)		Model2 OR (95%CI)		
Overweight defined by VFA							
Not obese	798(61.0%)	Ref.	Ref.		Ref.		
Obese	510(39.0%)	1.33* (1.07,1.66)	1.47* (1.15,1.86)		1.53* (1.19,1.96)		
Overweight defined by body mass index							
Not obese	700(53.5%)	Ref.	Ref.		Ref.		
Obese	608(46.5%)	0.95(0.76,1.17)		0.97(0.78,1.22)		0.97(0.77,1.23)	
Overweight defined by waist circumference							
Not obese	531(40.6%)	Ref.	Ref.		Ref.		
Obese	777(59.4%)	0.97(0.78,1.21)	0.96(0.76,1.20)		0.95(0.74,1.20)		

Model1 adjusted by sex and age

Model2 adjusted by sex, age, drinking, smoking, education, depression, widowed, living alone and chronic diseases (Diabetes, Stroke, Heart disease)

Overweight (BMI): BMI ≥ 24.0 kg/m^2^; Overweight (VFA): VFA ≥ 100m^2^; Overweight (WC): Men: WC ≥ 85 cm, Women: WC ≥ 80 cm

*P < 0.05

### The risk of prefrailty based on the combination of BMI and VFA

After categorizing participants based on the combination of BMI and VFA, it was showed that the risk of prefrailty was significantly higher among individuals with normal BMI but with a high VFA [OR (95% CI) of 1.76 (1.10,2.82)] and with obese BMI but with a high VFA [OR (95% CI) of 1.75(1.17,2.60)] after adjusting for sex and age compared to participants with normal BMI and normal VFA groups. Interestingly, those who were overweight but had a normal VFA [OR (95% CI) of 0.64(0.45,0.92)] have a lower risk of prefrailty compared to individuals with normal BMI and normal VFA. Adjusted for Model 2, the results remained statistically significant (Table 3).

**Table 3:** Pre-frailty risk according to BMI after taking into consideration of VFA

VFA (cm^2^)		BMI (kg/m^2^)			
		Underweight (< 18.5)	Normal (18.5–23.9)	Overweight (24.0-27.9)	Obese (≥ 28.0)
Unadjusted	Normal VFA	1.61(0.88,2.93)	Ref	0.64* **(0.45,0.90)**	1.05(0.30,3.67)
	***n*** events/total	29/48	287/560	68/180	5/10
	High VFA	–	1.31(0.84,2.04)	1.10(0.83,1.47)	1.54* **(1.05,2.26)**
	***n*** events/total	–	51/92	146/285	79/133
Model1	Normal VFA	1.70(0.91,3.18)	Ref	0.64* **(0.45,0.92)**	1.10(0.31,3.94)
	High VFA	–	1.76* **(1.10,2.82)**	1.19(0.88,1.60)	1.75* **(1.17,2.60)**
Model2	Normal VFA	1.84(0.96,3.52)	Ref	0.62* **(0.43,0.90)**	1.02(0.27,3.78)
	High VFA	–	1.87* **(1.15,3.03)**	1.25(0.91,1.70)	1.76* **(1.17,2.66)**

Model1 adjusted by sex and age

Model2 adjusted by sex, age, drinking, smoking, education, depression, widowed, living alone and chronic diseases (Diabetes, Stroke, Heart disease)

*P < 0.05

### The prevalence of prefrailty after taking into consideration VFA and BMI

**Fig. 1:**
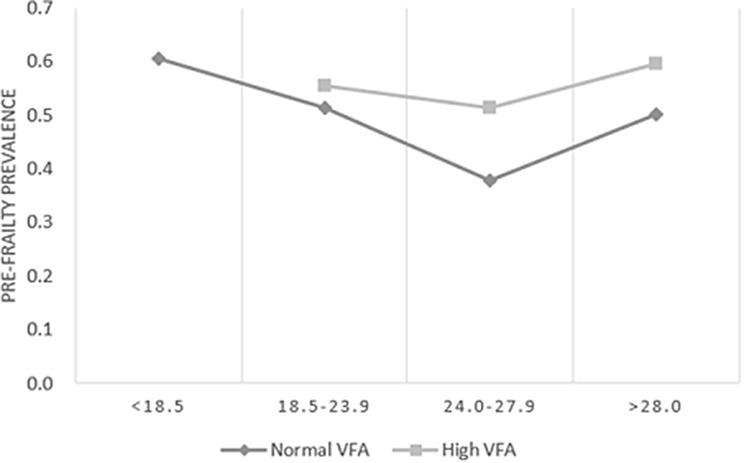
Association of BMI taking into consideration VFA with regard to the prevalence of pre-frailty

Overall, within the same BMI range, older adults with high VFA had a higher prevalence of prefrailty relative to those with normal VFA, and those with overweight BMI had the lowest prevalence of prefrailty regardless of high VFA. In addition, a higher prevalence of prefrailty was also shown in those with normal VFA but underweight BMI (Fig. 1).

## Discussion

Our research is the first of its kind to investigate the correlation between prefrailty and various indicators of obesity in older Chinese inhabitants of the community. We found that only visceral obesity was relevant to prefrailty, whereas other obesity indicators, even waist circumference, which also represents abdominal adiposity, were not associated with prefrailty. Interestingly, compared to those with both normal VFA and BMI, those with overweight BMI and normal VFA were negatively associated with prefrailty, meaning that overweight BMI and normal VFA were protective factors for prefrailty, whereas those with normal weight but excess visceral fat area had a higher risk of prefrailty. The findings of this study indicate a significant link between visceral adiposity and prefrailty among older adults residing in Chinese communities. The evaluation of visceral fat could hold considerable significance in the prevention of prefrailty.

### Visceral fat and prefrailty risk

Our study found that visceral fat was a risk factor for pre-frailty, not BMI or waist circumference. This suggests that we should focus more on changes in visceral fat in pre-frail older adults. A Japanese study [30] found that in community-dwelling older adults, visceral fat area was the only risk factor for prefrailty compared to body fat percentage and BMI, consistent with our findings. Their diagnostic approach for frailty and visceral fat was also consistent with ours. In contrast, Brazilian study discovered a positive association between waist circumference and prefrailty, contradicting our results. In older adults, whereas overweight was a protective factor for pre-frailty [12]. A study using segmented logistic regression found that among patients with advanced lung disease in the United States, those with high visceral adiposity were 50% more likely to have frailty for every 20 cm2 increase in VFA, whereas patients with low visceral adiposity had a 10% lower chance of frailty [31]. The results may be biased due to different racial criteria, inclusion of populations with different diagnostic criteria.

### Differences between visceral fat and waist circumference

There were some differences between visceral fat and waist circumference, which may explain why it was visceral fat rather than waist circumference that was more associated with pre-frailty. Waist circumference did not differentiate between subcutaneous and visceral fat, although there were associations between waist circumference and chronic inflammation, oxidative stress and insulin resistance [32, 33]. Of these, visceral fat is more clearly associated with insulin resistance [34], while subcutaneous fat has a protective effect on insulin sensitivity [35]. The amount of abdominal visceral adipose tissue is more closely linked to cardiometabolic risk factors such as fasting glucose, triglycerides, and HDL-cholesterol levels than subcutaneous adipose tissue [34]. Visceral adiposity mediates many pathogenic mechanisms, and these are strongly associated with the pathogenesis of frailty. Therefore, it may be understood that visceral fat obesity, which is strongly associated with insulin resistance and metabolic disturbances, is a better predictor of prefrailty than waist circumference. Because prefrailty can be reversed, it is crucial to comprehend the connection between visceral adiposity and prefrailty in order to prevent frailty in older persons. Through longitudinal investigations, more investigation is required to establish the cause-and-effect relationship between VAF and prefrailty.

### BMI and visceral Fat: combined impact on prefrailty

It was also found that overweight BMI with normal visceral fat reduced the prevalence of prefrailty by 38%, while those with normal weight but excess visceral fat area had a higher risk of prefrailty. This suggests that we need to consider the effect of BMI and VFA on prefrailty together. Alternatively, we need to consider the distribution and type of adipose area [20]. BMI is more directly associated to general obesity and weight, representing overall nutrition. And central obesity are more directly related to abdominal obesity and metabolic-related diseases (e.g., metabolic syndrome, diabetes, coronary heart disease, etc.) [36, 37], which are also part of the pathogenesis of frailty. Less visceral fat represents lower metabolic risk, while a slightly higher BMI can actually be shown a protective factor against malnutrition, fractures and cognitive decline [38]. Therefore, a combination of fat distribution and type may be able to more finely predict the onset and risk of prefrailty.

### The obesity paradox in older adults

Obesity is widely recognized for its role in promoting insulin resistance, oxidative stress, and inflammation, all of which can contribute to the development of frailty [13, 14]. Studies conducted in the past have revealed that the so-termed “obesity paradox” or “reverse epidemiological paradox” [39], which is a protective effect of obesity, is also seen in the context of frailty. The obesity paradox suggests that excessive fat is not always harmful. In this study, people with a BMI < 18.5 had the highest prevalence of prefrailty, with a prevalence of 60.42%. Underweight may be caused by chronic disease or malnutrition and sarcopenia, which are also associated with an increased risk of frailty [40]. In general, adiposity can provide energy reserves to protect against acutely stressful events, such as acute illness [15]. In the English Longitudinal Study of Ageing, an inverted J-shaped distribution of the association between BMI and frailty was observed among those with lower waist circumference, with the lowest prevalence of frailty among those who were overweight or obese [18]. Our study also found that the prevalence of prefrailty was lowest for those with a BMI in the overweight range, regardless of whether they had visceral fat obesity. A systematic evaluation and meta-analysis also showed that the BMI with the lowest risk of death in older adults is actually represented by overweight or even mild obesity [41, 42]. However, it should be clarified that while a higher BMI may be protective for older adults, this does not negate the excessive adiposity can reduce the ability of older adults to engage in physical activity and increase metabolic instability, which can lead to frailty [43]. The correlation between BMI and general obesity in younger and middle-aged adults has been demonstrated, yet BMI does not account for the fact that older individuals tend to decrease in height as they age [44]. Thus, the BMI range for overweight adults is a normal BMI range for older adults, which may explain the lower risk of frailty and mortality in overweight older adults.

### Strategies about prevent prefrailty

Prefrailty was prevalent in the current study at 49.7%, which was significantly higher than a previously reported prefrailty prevalence rate [3], implying that the identification and prevention of prefrailty is of greater concern. With the application of technology in the medical field, real-time monitoring of body composition data allows for prompt assessment of obesity type, which in turn forecast and monitors the development of frailty and supports the reversal of pre-frailty. In addition, multicomponent and resistance training programs have proven effective in preventing and reversing prefrailty and frailty among community-dwelling middle-aged adults [45]. Moreover, it is necessary to further explore interventions aimed at reducing visceral fat, such as a healthy diet habit [46]and aerobic exercise [47], which may be beneficial in reversing the progression to frailty.

### The limitations of the current research

The current research has some limitations. First, the causal association between VFA and frailty risk could not be used to determine the cross-sectional study design. Second, the sample size for this study was somewhat small, especially when BMI and VFA were considered together; for example, too few people met the criteria for both high BMI and normal VFA, which to some extent could cause bias in the statistical results. And we did not investigate dietary habits which are an important factor in visceral lipogenesis. Moreover, there are additional potential sources of bias. Our study population was relatively healthy, as it included older adults capable of traveling from home to community or hospital settings, indicating a selection bias towards healthier individuals. To address these limitations and biases, future research should aim to increase the sample size and include a more diverse and representative cohort of older adults. Conducting longitudinal studies will also be crucial in validating our conclusions and better understanding the causal relationships between visceral fat obesity and prefrailty.

## Conclusion

The current study suggests that visceral fat obesity is an independent risk factor for prefrailty in this population. Consideration of VFA is important to prevent prefrailty in older adults in the community. Implementing targeted interventions, such as dietary modifications, increased physical activity, and other lifestyle changes, may play a crucial role in reducing the risk of prefrailty and improving overall health outcomes in this population.

## Data Availability

The raw data supporting the conclusions of this article will be made available by the authors, upon reasonable request. Requests to access the datasets should be directed to the corresponding author (guoqijp@gmail.com).
